# Validation of the Portuguese Version of the Risk Instrument for Screening in the Community (RISC) Among Older Patients in Primary Care in Northern Portugal

**DOI:** 10.3389/fpubh.2021.614935

**Published:** 2021-08-13

**Authors:** Sara Santos, Rónán O'Caoimh, Laetitia Teixeira, Sara Alves, William Molloy, Constança Paúl

**Affiliations:** ^1^Abel Salazar Institute of Biomedical Sciences, University of Porto, Porto, Portugal; ^2^CINTESIS, Faculty of Medicine, University of Porto, Porto, Portugal; ^3^Centre for Gerontology and Rehabilitation, St Finbarrs Hospital, University College Cork, Cork, Ireland

**Keywords:** primary care, older people, death, hospitalization, institutionalization, risk

## Abstract

**Background:** Aging is associated with an increase in adverse health outcomes for older people. Short screening instruments that easily and quickly identify those at highest risk can enable decision-makers to anticipate future needs, allocate scarce resources and act to minimize risk. The Risk Instrument for Screening in the Community (RISC) is a brief (2–5 min) Likert scale that scores one-year risk of institutionalization, hospitalization and death from low (1/5) to severe (5/5).

**Objectives:** To externally validate the RISC, scored by general practitioners (GP's), in primary care in Northern Portugal.

**Methods:** The RISC was translated and culturally adapted to Portuguese. A cohort of 457 older adults (aged ≥65) under active follow-up with their GP's were screened. Outcomes at one-year were recorded. Accuracy was determined from the area under the curve (AUC) of receiver operating curve analysis.

**Results:** The mean age of participants was 75.2 years; 57% were female. The proportion identified as being at maximum risk (RISC scores of 3-5/5) of institutionalization, hospitalization and death, were 14.9, 52.4 and 38.4%, respectively. At follow-up 2% (10/431) were institutionalized, 18.6% (84/451) were hospitalized and 3% (14/456) died. Those who were institutionalized (*p* = 0.021), hospitalized (*p* = 0.012) or dead (*p* < 0.001) at one-year were significantly older. Those living alone were more likely to be institutionalized (*p* = 0.007). The RISC showed fair accuracy in predicting hospitalization (AUC of 0.62 [95% CI: 0.55–0.69]) and good accuracy for Institutionalization (AUC of 0.79 [95% CI: 0.62–0.96]) and death (AUC of 0.77 [95% CI: 0.65–0.88]).

**Conclusions:** The Portuguese version of the RISC accurately predicted institutionalization and death at one-year but like most short screens was less able to predict hospitalization. Given its brevity, the RISC is useful for quickly identifying and stratifying those at increased risk in primary care.

## Introduction

Population aging will have implications in almost every sector of society: labor, finances, social security, and healthcare services. These demographic changes are most evident in Europe where in 2050 it is expected that 35% of the population will be more than 60 years old (1). In the European Union (EU) the proportion of the population aged over 65 years will increase from 19.8 to 31.3% in the EU-28 countries between 2018 and 2100 (2). Portugal has one of the oldest populations in the EU (3). The economic crisis experienced in the last decade and social changes caused by the migration of younger people, is creating rapid population aging throughout Portugal (4). The average life expectancy of the Portuguese population is slightly above the EU average, with women living on average 6.2 years longer than men. However, more than half of all the years lived by Portuguese adults after 65 (20.4 years) are lived with some level of disability (13.1 years) (5).

Often associated with aging and disability, frailty is a multi-dimensional construct that increases risk of adverse outcomes (6). It has a high incidence and prevalence in EU countries including Portugal (7, 8). Multimorbidity, defined as the increased risk of having several diseases that coexist simultaneously (9), is likewise age-associated. In Portugal, half (53%) of persons have at least one chronic disease and almost one-fifth (17%) have two or more (10). Several studies indicate that health inequalities such as low socioeconomic status are associated with multimorbidity (9), affecting function, quality of life and risk of mortality (11). These in turn increase demand for health services and associated costs (12). Health inequalities are common in Portugal (13, 14) and it is expected that these will be amplified in an aging population, placing added pressure on already stretched healthcare systems (15).

In a recent review of the Portuguese National Health System (NHS), regional inequalities were identified as an additional factor with regions affected most by population aging lacking resources (doctors, nurses, beds, health units), further limiting access to adequate healthcare (4). Integrated into the NHS, primary care services (PC) are the first line of intervention and are recognized as the cornerstone of any health system. In Portugal they provide a comprehensive and continuous service, with a focus on the person throughout their life course, which makes it possible to identify causes and risk factors, providing an appropriate response to different health situations (16). To better allocate scarce economic and human resources, healthcare professionals need to have objective information regarding the older patients they serve in order to best target certain clinical services to those who would benefit the most (17). Within PC, the General Practitioner (GP) has a fundamental role, as a point of first contact with the NHS, in monitoring acute and chronic problems and in gatekeeping referrals to specialist health care (18). GPs maintain close relationships with their patients and may be best placed to help identify community-dwellers at risk of adverse events.

Risk assessment using risk-prediction models provide data and information to support decision-making. These not only consider the magnitude and likelihood of risk, but also the underlying costs and benefits of managing this risk (19). A variety of different tools are used to identify older adults at risk of adverse healthcare outcomes, to guide healthcare professionals and individuals in their decision-making process (20). Most are too long, have poor predictive ability, do not adequately stratify risk or assess the ability of older persons' caregiver networks to manage risk (21). To accurately determine the requirement for appropriate interventions, it is essential to stratify and comprehensively assess older adults, taking into account their supports including their social (caregiver) network (22). The Risk Instrument for Screening in Community (RISC) is a screening instrument that assesses the risk of adverse outcomes in community-dwelling older adults across three main domains: mental state, activities of daily living (ADL) and medical state, taking into account the ability of the social network to manage the individual's needs (23). The RISC is a brief and subjective risk-prediction instrument, developed to assess the risk perceived by healthcare professionals that community-dwellers will be hospitalized, institutionalized or die at 1 year (23). It is validated in English-speaking countries (Ireland, the UK, and Australia), Spain and Portugal (24).

Given the importance and the need for valid and reliable instruments for the rapid and effective identification of people at risk of adverse outcomes, this paper, a sub-study of a larger study carried out in Northern Portugal, aims to examine the predictive validity of the Portuguese version of RISC, scored by GP's in a sample of primary care patients aged ≥65, for three adverse outcomes: hospitalization, institutionalization and death, after 1 year.

## Methods

### Design and Participants

This paper is part of a larger study, conducted in the Northern Portuguese Health Primary Care Services between 2014 and 2016. The project, developed in accordance with the Declaration of Helsinki, was approved by the ethics committee of the Regional Association of Health North and by each of the Associations of Health Centers in the region where data were collected (25). The larger research project includes Portuguese people aged ≥65 years, who live in the community and were patients of PC in the area covered by the Portuguese North Region Health Authority (ARS North). Individuals aged <65, those who were not under regular follow-up with their GP, who were institutionalized (i.e., nursing home residents) and who were at the end-of-life (including those receiving palliative care) were excluded. As this study will evaluate the ability of primary care to predict adverse outcomes through the application of the RISC, this sub-sample included only participants who were screened by their GP and had one-year follow-up data available.

### Measures and Procedures

The RISC is a short (administration time of 2–5 min) screening instrument that identifies older people at risk of adverse events. It collects demographic data and identifies perceived concern across 3 domains: Mental State, ADL State, and Medical State. The risk in each domain is identified as present or absent and the ability of the caregiver network (including services) to respond to situations identified in the domains is also considered and scored from 1 (can manage) to 5 (absent/liability). Each domain is evaluated by its degree of severity, from mild to severe, and the availability and management capacity of the support network. The risk is determined in this way: Risk equals the severity level less the protective effect of the support network. In the end, based on the assessment, three subjective 5-point Likert scales called *Global Risk Levels* are scored from 1 (low risk) to 5 (high risk), indicating the perceived probability of adverse events (institutionalization, hospitalization or death), within 1 year (24, 26). Developed by University College Cork (UCC), Ireland in English (27), the RISC was validated in Ireland (23) and has shown fair to good accuracy in predicting institutionalization, hospitalization and death over a 1 year period, has good to excellent reliability and internal consistency (28). The RISC scoring sheet is available in English at: https://bmcgeriatr.biomedcentral.com/articles/10.1186/1471-2318-14-104/figures/1.

The translation back-translation method was used to develop the Portuguese version of the RISC, which is linguistically correct and equivalent semantically to the original version. A committee of three experts in gerontology, all fluent in English, translated the instrument to Portuguese and that version was back-translated to English by a professional English translator and by a professional with experience in gerontology. The result was discussed with the team of authors and some minor changes were made.

The final version of the instrument was scored by healthcare professionals in primary care including GP) and practice nurses, who agreed to participate scoring only their own patients, whom they knew well after reviewing their records. Four hours of training on how to score the RISC was provided. This was delivered by the research team in the local health-care units. Training sessions play an important role in increasing inter-rater reliability for the RISC (29). The content covered in the training was based on the two-day training sessions that the research team in UCC provided. Concepts related to risk and the adverse outcomes, frailty and multi-morbidity, as well all the contents of the instrument, were discussed and analyzed, with a special focus on how the care network can influence the overall risk assessment (30).

Adverse outcomes within the next 12 months were reported by the GPs based on their available records. Institutionalization was defined when patients entered into a nursing home for a permanent stay, hospitalization referred to acute admissions to a hospital.

### Statistical Analysis

Data were analyzed with SPSS (26.0). Descriptive statistics were used for the characterization of the sample and risk profile identified by GPs at screening. In order to facilitate the analysis, we dichotomised the *Global Risk Scores* into minimum risk (scores 1–2) and maximum risk (*Global Risk Scores* 3–5) (24). Group analysis comparisons were performed using the Mann–Whitney *U* test for continuous variables and Chi-square test for categorical variables (significance level of 0.05). Accuracy was determined from the area under the curve (AUC), calculated from receiver operating characteristic (ROC) curves, for each outcome and components of the instrument such as the caregiver network score. AUC values between 0.5–0.59 were taken as a fail, 0.60–0.69 as poor, 0.70–0.79 as fair and >0.8 as good to excellent. Optimal cut-off scores were obtained using Youden's Index (i.e., Sensitivity – Specificity + 1).

## Results

The sample comprised 457 individuals who were screened with the RISC. The mean age of those included was 75.16 years, (SD: +/– 6.82), range 65–97 years. In all, 193 participants were male (42%) and 264 (58%) female. In total, 14.4% (66) lived alone. Of the remainder, 85.6% (391), 319 lived with their spouse, 49 with their children and 19 with other family members. Regarding the concerns identified by GPs, most, 447 (97.8%), raised medical concerns. The majority, 325 (71.0%) also raised ADL concerns and mental health concerns, 317 (69.5%) (Table 1). Regarding the severity of the concerns identified, the majority were rated as moderate to severe, irrespective of domain. Examining the data for the perceived ability of the caregiver network to manage the concerns identified, GPs perceived that the majority could manage (as was needed) medical concerns (*Can manage*−53.7%). Higher proportions of patients' caregiver networks had perceived difficulties managing the ADL's and mental health concerns of the patients. None of the patients lacked a network where it was deemed necessary.

**Table 1:** Proportion of patients raising Mental Health, ADL's, and Medical Concerns on the Risk Instrument for Screening in Community (RISC) including the perceived severity level and ability of the caregiver network to manage concerns.

**Mental Health Concerns**								
	*N*	%						
No	139	30.5						
Yes	317	69.5	Severity			Caregivers ability to Manage		
				*N*	%		*n*	%
			Mild	136	43.0	Can Manage	147	46.7
			Moderate	150	47.5	Carer Strain	106	33.7
			Severe	30	9.5	Some Gaps	51	16.2
						Cannot Manage	11	3.5
						Absent/liability	0	0
**ADL's Concerns**								
	*N*	%						
No	132	28.9						
Yes	325	71.0	Severity			Caregivers ability to Manage		
				*N*	%		*n*	%
			Mild	61	18.8	Can Manage	120	36.9
			Moderate	182	56.0	Carer Strain	133	40.9
			Severe	82	25.2	Some Gaps	56	17.2
						Cannot Manage	16	4.9
						Absent/liability	0	0
**Medical Concerns**								
	*N*	%						
No	10	2.2						
Yes	447	97.8	Severity			Caregivers ability to Manage		
				*n*	%		*n*	%
			Mild	118	26.5	Can Manage	240	53.7
			Moderate	238	53.4	Carer Strain	137	30.6
			Severe	90	20.2	Some Gaps	57	12.8
						Cannot Manage	13	2.9
						Absent/liability	0	0

**Figure 1:**
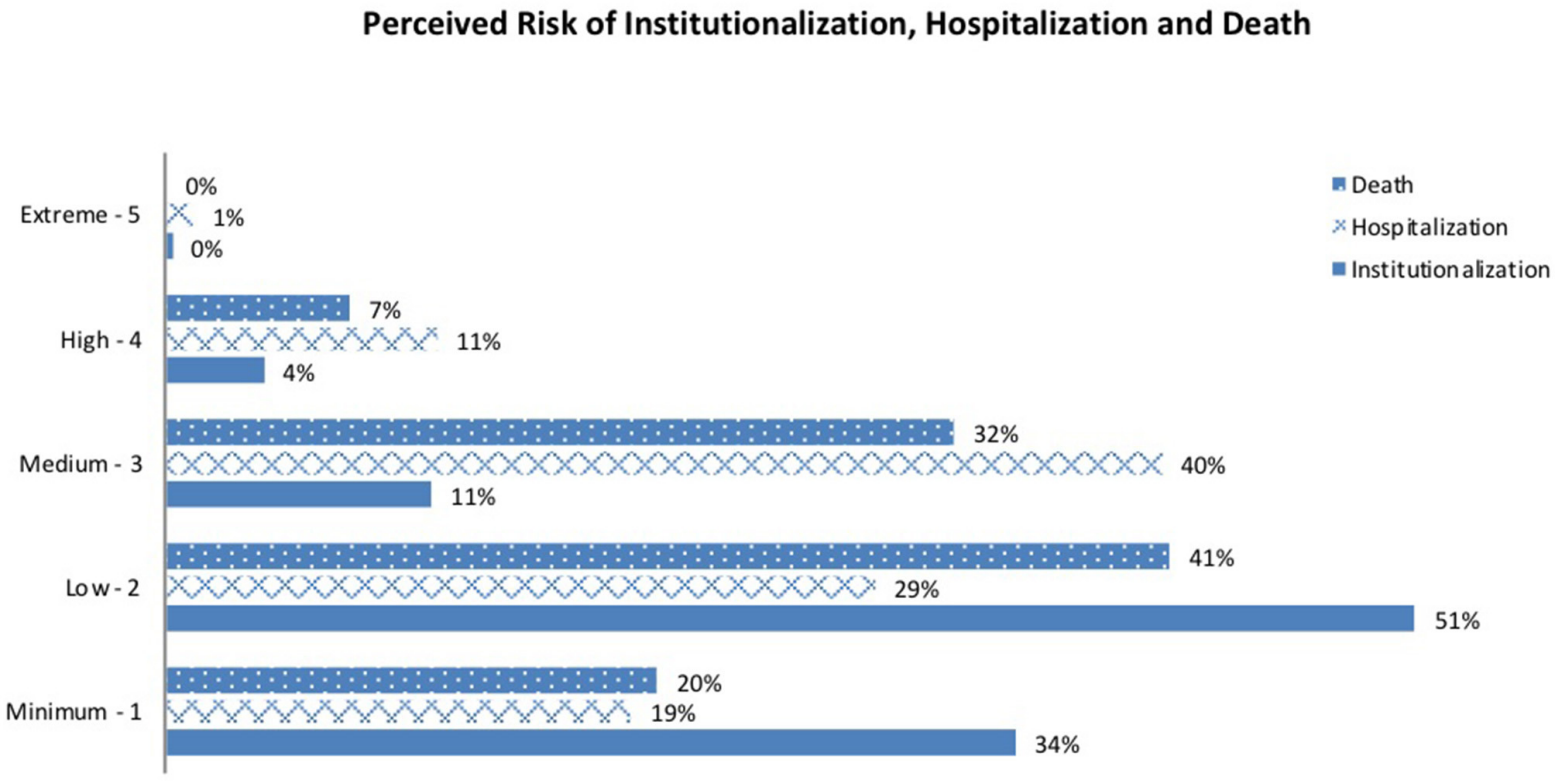
Proportion of patients according to their perceived one-year risk (*Global Risk Scores*) of institutionalization, hospitalization, and death as scored by the general practitioner.

The overall (perceived) risk scores (*Global Risk Scores*) for each of the three in the following year (i.e., of being institutionalized, hospitalized or dying) were then assessed after completing the scoring of the three domains. Based on this, GP's, rated 14.9% (18) of participants as being at maximum risk of institutionalization, 52.4% (239) were perceived to be at maximum risk of hospitalization and 38.4% (180) were scored at maximum risk of death at one-year. Few patients if any, were scored as extreme (certain) risk. These data are presented in Figure 1.

### One-Year Adverse Outcomes—Proportion and Predictors

One year later (Table 2), the occurrence of each of the three possible adverse events was assessed. The proportion institutionalized was approximately 2% (*n* = 10), while 18.6% (*n* = 84) were hospitalized and 3% (*n* = 14) were dead.

**Table 2:** Comparison of patient characteristics by adverse outcome after one-year.

	**Institutionalized**	**Not institutionalized**	***p-value***	**Hospitalized**	**Not hospitalized**	***p-value***	**Death**	**Alive**	***p-value***
Age Mean (Sd)	*n = 10 (2%)*	*n = 431 (98%)*		*n = 84 (19%)*	*n = 367 (81%)*		*n = 14(3%)*	*n = 442(97%)*	
	80.50	74.78	0.008^*^	76.84	74.75	0.059	84.21	74.88	0.000^*^
Gender	*n = 10*	*n = 431*		*n = 84*	*n = 367*		*n = 14*	*n = 442*	
Female	6 (60.0%)	252 (58.4%)	0.92	41 (48.8%)	221 (60.2%)	0.56	5 (35.7%)	259 (58.6%)	0.88
Male	4 (40.0%)	179 (41.5%)		43 (51.2%)	146 (39.8%)		9 (64.3%)	183 (41.4%)	
Living Arrangements	*n = 10*	*n = 428*		*n = 84*	*n = 364*		*n =13*	*n = 439*	
Alone	5 (50.0%)	60 (14.0%)	0.007^*^	10 (11.9%)	56 (15.4%)	0.70	1 (7.7%)	65 (14.8%)	0.78
With others	5 (50.0%)	368 (85.9%)		74 (88.1%)	308 (84.6%)		12 (92.3%)	374 (85.2%)	

* *Statistically significant with P < 0.010*.

Those who were older (*p* = 0.008) and living alone (*p* = 0.007) were statistically significantly more likely to be institutionalized. Those who were older were also more likely to die within 1 year (*p* < 0.001). There was no association found between gender and any of the outcomes.

### Predictive Accuracy of the RISC

**Figure 2:**
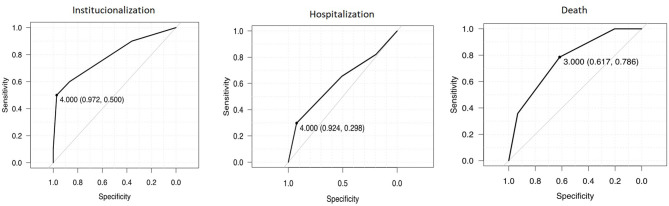
Receiver Operating Characteristics curve demonstrating sensitivities and specificities for the Risk Instrument for Screening in The Community in identifying one-year risk of institutionalization, hospitalization, and death.

Figure 2 presents ROC curves showing the accuracy of the RISC to predict institutionalization, hospitalization and death. The AUC scores obtained show that the RISC had poor accuracy in predicting hospitalization (AUC of 0.62 [95% CI: 0.55–0.69]), but fair to good accuracy in predicting Institutionalization (AUC of 0.79 [95% CI: 0.62–0.96]) and death (AUC of 0.77 [95% CI: 0.65–0.88]).

Based upon the sensitivity and specificity obtained from the ROC curve analysis, the optimal cut-off score on the RISC for hospitalization was ≥4, giving a poor sensitivity of 30% but high specificity of 92%. The optimal cut-off point for institutionalization was ≥4 with a sensitivity of 50% and specificity of 97% and an optimal cut-off score ≥3 with a sensitivity of 79% and specificity of 62% to predict death. These values were similar when Youden's Index was applied (Table 3), though a cut-off of ≥3 provided a better balance between sensitivity (60%) and specificity of (87%) for hospitalization.

**Table 3:** Sensitivity, specificity, and Youden's Index values for the Risk Instrument for Screening in The Community (RISC) for predicting outcomes for of the sample after one-year.

**Cut-off score on the RISC**	**Outcome**	**Sensitivity**	**Specificity**	**Youden's Index**
≥2	Institutionalization	90%	36%	0.26
	Hospitalization	82%	19%	0.01
	Death	100%	0%	0.00
≥3	Institutionalization	60%	87%	0.47
	Hospitalization	65%	51%	0.16
	Death	79%	62%	0.41
≥4	Institutionalization	50%	97%	0.47
	Hospitalization	30%	92%	0.22
	Death	36%	93%	0.29
≥5	Institutionalization	0%	100%	0.00
	Hospitalization	2%	99%	0.01
	Death	NA	NA	NA

Taking into account that the RISC includes several components/parts that contain information about the 3 domains as well the functioning of the caregiver network, it is important to understand if any of these sub-components were more accurate in predicting outcomes than the *Global Risk Score*. The accuracy of each component of the RISC to predict the results at one-year are presented in Table 4. The presence or absence of *Concern* on its own was at best a poor predictor of hospitalization, irrespective of domain assessed. It also showed poor predictive ability for institutionalization with an AUC of 0.65 (CI: 0.52–0.78) for ADL's and 0.66 (CI: 0.55–0.77) for death related to mental health.

**Table 4:** Area under the receiver operating characteristic curve scores with 95% confidence interval for the *Global Risk Scores* and components of the RISC scores including: mental state, activities of daily living (ADL) and medical state domains and caregiver network.

	**Outcomes after 1-year**		
**Variable**	**Institutionalization**	**Hospitalization**	**Death**
**RISC Global Risk score**	0.79 (0.62–0.96)^**^	0.62 (0.55–0.69)^**^	0.77 (0.65–0.88)^**^
Mental State			
*Concern*	0.61 (0.46–0.76)	0.56 (0.49–0.62)	0.66 (0.55–0.77)^*^
*Severity of concern*	0.76 (0.59–0.92)^**^	0.64 (0.56–0.72)^**^	0.63 (0.45–0.81)
*Caregiver Network*	0.77 (0.61–0.94)^**^	0.68 (0.60–0.75)^***^	0.68 (0.54–0.82)^*^
ADL's			
*Concern*	0.65 (0.52–0.78)	0.58 (0.52–0.65)^*^	0.61 (0.48–0.74)
*Severity of concern*	0.65 (0.44–0.86)	0.64 (0.56–0.73)^**^	0.73 (0.60–0.86)^**^
*Caregiver Network*	0.63 (0.43–1.84)	0.63 (0.55–0.70)^**^	0.66 (0.53–0.79)^*^
Medical State			
*Concern*	0.51 (0.33–0.69)	0.51 (0.45–0.58)	0.51 (0.36–0.66)
*Severity of concern*	0.80 (0.68–0.92)^**^	0.67 (0.59–0.75)^***^	0.73 (0.60–0.86)^**^
*Caregiver Network*	0.74 (0.57–0.91)^*^	0.68 (0.61–075)^***^	0.63 (0.48–0.78)

* *Statistically significant with P < 0.05*.

** *Statistically significant with P < 0.01*.

*** *Statistically significant with P < 0.001*.

The *severity* of the concern however, appeared to be a better predictor for institutionalization with good accuracy, an AUC of 0.80 (CI: 0.68–0.92) for the patients' medical state and fair accuracy, 0.76 (CI: 0.59–0.92) for their mental state. The ability of *caregiver networks'* more accurately predicted institutionalization (AUC: 0.77 [CI: 0.91–0.94]) than hospitalization or death, both with an AUC of 0.68. Although the Global RISC score had poor accuracy in predicting hospitalization (AUC of 0.62 [95% CI: 0.55–0.69]), its sub-components showed slightly higher accuracy i.e., the AUC values for *severity of concern* and the *caregiver network* showed higher predictive values in all three domains, though these were not significantly different with overlapping confidence intervals.

## Discussion

This sub-study of a larger study carried by Paul et al. (30), analyzed the accuracy of the RISC to predict the occurrence of three adverse outcomes at one-year: institutionalization, hospitalization and death in a sample of community-dwelling older adults aged ≥65 in Northern Portugal, based on GP's perception of their risk. The RISC incorporates concerns across in three domains: mental, ADL (functional) and medical balanced against the ability of the caregiver network (where required) to assess risk.

In this sample, there was most concern over medical state issues (97.8%) followed by concerns over ADL function (71%) and mental state (69.5%). Based on the RISC assessment, 14.9, 52.4, and 38.4% were scored as maximum risk (RISC Score 3–5) of institutionalization, hospitalization and death, respectively.

These proportions are comparable to findings in other studies. In Ireland, O'Caoimh at al. (24) found similar risk profiles in 803 community dwelling older adults (≥65). In that study 12.3, 36.2, and 20.6% were at maximum risk of institutionalization, hospitalization and death, respectively. Of a sample of 4,499 community-dwellers aged ≥65 living in the North of Portugal, screened with the RISC, 16.3% at maximum risk of institutionalization, 32.8% of hospitalization and 23.1% of death (30). While similar, data from Portugal identifies a slightly higher level of risk when compared with the Irish study. In Portugal, 60% of older people live alone or with persons with more than 65 years old (INE, 2012) potentially increasing their risk of adverse outcomes like hospitalization, institutionalization and death (31) referred above. Comparing these studies with another sub-study by Brandão at al. (32), scoring the RISC on Portuguese patients aged ≥80 exclusively with mental health concerns, also showed higher rates of perceived risk, indicating that context and additional risk factors can further increase RISC scores. As expected these patients also had a correspondingly higher incidence of adverse outcomes than that found in this study (30). At one-year the proportion institutionalized, hospitalized and dead were 12.1, 25.2, and 19.0% respectively. In the current study, after one-year, the proportion of patients that were institutionalized, hospitalized and dead was markedly lower at 2, 19, and 3% respectively.

Factors such as age, social isolation, co-morbidity levels and the area of residence are identified in other studies as predictors of hospitalization (33). Of these, none of the variables is statistically significant for hospitalization. Age and living arrangement (living alone) were associated with institutionalization. Among older adults aged ≥75 years, the probability of institutionalization increased significantly with a reduced caregiver network (e.g., widowhood; OR = 78.3), mental health issues (e.g., dementia; OR = 154.1) and ADL concerns (e.g., impaired mobility; OR = 36.7) (34), all commonly identified concerns identified in this study and others examining the care needs of older people (35).

This study showed that RISC had variable accuracy in predicting all three adverse outcomes. It had fair to good accuracy in predicting institutionalization (AUC of 0.79) and death (AUC of 0.77), but poor accuracy in predicting hospitalization (AUC of 0.62). O'Caoimh at al. (28) found similar results for hospitalization among community dwellers in Ireland (AUC of 0.61). This fact may reflect the complexity associated with the prediction of hospitalization in an imminently fragile population, very susceptible to the occurrence of exacerbations of their multiple chronic diseases (36–38), which makes the GP's assessment more difficult. However, when we analyse the components of RISK, the severity of the medical state [AUC of 0.67–*P* < 0.001] and the ability of the caregiver network (AUC of 0.68–*P* < 0.001) to manage them had demonstrated a better ability to predict hospitalization than the global risk score. The severity of the illness and the ability of the care network to manage it may trigger the aggravation of chronic pathologies, leading to episodes of hospitalization. Similarly, the caregiver network ability to manage de mental state (AUC of 0.68–*P* < 0.001) was more accurate predicting hospitalization than the global risk score for this outcome. According to Bernardes at al., dementia patients represent a significant amount of hospital admission in mainland Portuguese public hospitals (39), fact that could be a consequence of the ability to manage, or not, the mental state concern. This data could be used by GP's to target the main factors that may point to an increased risk of hospitalization making it possible to identify and provide timely intervention to minimize them.

The RISC showed good accuracy in the prediction of institutionalization [AUC of 0.79 (95% CI: 0.62–0.96)]. This is also consistent with previous studies, reinforcing the good predictive ability of RISC for institutionalization (36). When analyzing the components of the test we identify that, in our study, the severity of medical status (AUC of 0.80) was particularly accurate in identifying those likely to be institutionalized. Among the many factors associated with institutionalization, functional decline as well as the capacity to perform ADL usually appear as strong predictors of institutionalization (24, 40). The results also suggest that the severity of mental health concerns is a good predictor of institutionalization [AUC of 0.76 (95% CI: 0.54–0.92)]. These data are corroborated by a study conducted by Vandepitte at al. where caregiver burden among carers of people with dementia, especially in dealing with behavioral disorders, is associated with the desire to pursue long-term care (41). In this study we showed that the caregiver network's ability to cope with mental health problems can also predict institutionalization [AUC of 0.77 (95% CI:0.61–0.94)]. Again, this has been shown when the caregiver network score, a component of the RISC, has been studied alone (42). A study conducted in two French communities over 22 years concluded that the presence of informal caregivers, especially a partner, has a strong protective effect, reducing the risk of institutionalization by 40% for a person aged ≥80 (43). In our sample, approximately 8% (five out of 60) of the participants living alone were institutionalized compared with only 1.4% (five out of 368) of those living with others. However, although living alone is a risk for adverse outcomes such as institutionalization, according to Sakurai at al. older people who live with other family members and have poor social networks (few interactions with other people) are more likely to have health problems than other adults with good social networks who live alone (44). These data reinforce that a “good” social network is a protective factor for adverse events (42).

The RISC also had fair predictive validity for death [AUC of 0.77 (95% CI: 0.65–0.88)]. The severity of medical and ADL's concerns were also able to predict the occurrence of death (AUC of 0.73). This reflects results obtained by Teixeira et al. who identified the severity of medical concerns as the best predictive factor for the risk of death as perceived by GPs (45). The same study showed that if the caregiver network is unable to deal with medical problems at home, the perception of the probability of death within a year by GPs is 65 times higher for those at maximum risk compared those classified as lower risk. The association between the severity of ADL concerns and mortality has been thoroughly studied by others; the greater the severity and limitation in performing ADL's, the lower the life expectancy. Using a five stage scale (0—no limitations to 5—severe limitations) to evaluate the severity of ADL's, researchers have shown that the risk of death at 1 year increases five-fold if severe limitations in ADLs are present (HR = 5.2; 95% CI, 3.4–8.1) (46). It should be noted that after 1 year, only 3% of our sample (*n* = 14) had died. Their average age was 84 years, almost 10 years older than those alive at follow-up (75 years). More men (5%) than women (2%) died. In our study, age but not gender was significantly associated with death. Kusumastuti et al. based on a secondary data analysis of the Survey of Health, Aging, and Retirement in Europe (SHARE) identified age as the main predictor for one-year mortality with an AUC of 0.798 (95% CI 0.775–0.820) (47). Similarly, most instruments designed to predict death in community dwellers have only fair diagnostic accuracy with AUC values usually <0.80 [18].

Although this study has some strengths including the number of participants, there are some limitations. Our sample may not be representative as sampling was non-probalistic. Instead, it depended on the willingness of the medical teams to participate in the study, and to provide accurate follow-up data after 1 year. This may have introduced selection and reporting bias. To minimize this, patient selection was random. This study is based on a larger study carried out in the entire northern region of Portugal and thus the variables studied are restricted to those initially included. Hence, it was not possible to compare the diagnostic accuracy of the RISC with other short risk-prediction instruments or frailty screens. Another limitation is the information available to GPs, which may have limited their ability to evaluate the role of the caregiver network as a whole. Although GPs have a close relationship with their patient in clinical situations, the perception of the available supports (formal and informal) is more complex to identify and this may mean that RISC scores over or under-predicted one-year risk of each adverse outcomes affecting the accuracy of the RISC in this sample. A multidisciplinary assessment with the inclusion of other professionals may compensate for the lack of information on caregivers network, environment and socio-economic conditions.

## Conclusions

With an increasingly aging population, screening instruments that can easily and quickly identify and stratify older patients at risk of adverse outcomes will enable decision-makers in different areas of society to anticipate future needs, allocate scarce resources to those who need them most and ultimately act to minimize risk. This will likely result in better care and has the potential to lower costs (48). The RISC was most accurate in predicting institutionalization and death but as with other instruments and studies validating the RISC in other settings, it has poor predictive validity for hospitalization. This suggests that qualitative judgements made by healthcare professionals, with detailed knowledge of their own population, are a useful adjunct in assessing and potentially managing risk [20]. The existence of specific mental health, physical or ADL concerns is not sufficient to predict adverse outcomes. However, the ability of the caregiver network to manage these seemed to improve accuracy. However, more study is required to confirm this and the utility of using a subjective risk measure such as the RISC in routine clinical practice (i.e., outside of research settings). Nevertheless, the RISC appears to be a useful tool for the early identification of those at risk, potentially streamlining the assessment by utilizing existing clinician knowledge.

## Data Availability Statement

The datasets presented in this article are not readily available because belongs to a database still under analysis. Requests to access the datasets should be directed to Sara Santos, josefina.sampaio@gmail.com.

## Ethics Statement

The studies involving human participants were reviewed and approved by The Ethics Committee of the Regional Association of Health North - Portugal. The patients/participants provided their written informed consent to participate in this study.

## Author Contributions

SS, CP, and WM conceived of the present idea. SS wrote the manuscript in consultation with CP, RO'C, and WM. SS performed the analysis of data with input of RO'C. SS, LT, and SA contributed to the implementation of the research in the field. CP encouraged SS to investigate and supervise the findings of this work. All authors approved the final manuscript.

## Conflict of Interest

The authors declare that the research was conducted in the absence of any commercial or financial relationships that could be construed as a potential conflict of interest.

## Publisher's Note

All claims expressed in this article are solely those of the authors and do not necessarily represent those of their affiliated organizations, or those of the publisher, the editors and the reviewers. Any product that may be evaluated in this article, or claim that may be made by its manufacturer, is not guaranteed or endorsed by the publisher.
